# Maternity protection for female non-standard workers in South Africa: the case of domestic workers

**DOI:** 10.1186/s12884-022-04944-0

**Published:** 2022-08-22

**Authors:** Catherine Pereira-Kotze, Tanya Doherty, Mieke Faber

**Affiliations:** 1 School of Public Health, University of the Western Cape (UWC), Cape Town, South Africa; 2 Health Systems Research Unit, South African Medical Research Council (SAMRC), Cape Town, South Africa; 3 Non-Communicable Diseases Research Unit, South African Medical Research Council (SAMRC), Cape Town, South Africa; 4 Department of Dietetics and Nutrition, University of the Western Cape (UWC), Cape Town, South Africa

**Keywords:** Maternity protection, Non-standard workers, Domestic workers, Policy analysis, South Africa

## Abstract

**Background:**

Many women work in positions of non-standard employment, with limited legal and social protection. Access to comprehensive maternity protection for all working women could ensure that all women and children can access health and social protection. This study aimed to describe the maternity protection benefits available to women in positions of non-standard employment in South Africa, using domestic workers as a case study.

**Methods:**

A qualitative descriptive study design was used. National policy documents containing provisions on maternity protection were identified and analysed. Interviews were conducted with purposively selected key informants. Data extracted from published policy documents and information obtained from interviews were triangulated. A thematic analysis approach was used for evaluation of policy content and analysis of the interviews.

**Results:**

Twenty-nine policy and legislative documents were identified that contain provisions on maternity protection relevant to non-standard workers. These documents together with three key informant interviews and two media releases are used to describe availability and accessibility of maternity protection benefits for non-standard workers in South Africa, using domestic workers as a case study. Maternity protection is available in South Africa for some non-standard workers. However, the components of maternity protection are dispersed through many policy documents and there is weak alignment within government on maternity protection. Implementation, monitoring, and enforcement of existing maternity protection policy is inadequate. It is difficult for non-standard workers to access maternity protection benefits, particularly cash payments. Some non-standard workers have unique challenges in accessing maternity protection, for example domestic workers whose place of work is a private household and therefore difficult to monitor.

**Conclusion:**

The heterogeneity of non-standard employment makes it challenging for many women to access maternity protection. There are policy amendments that could be made and improvements to policy implementation that would enhance non-standard workers’ access to maternity protection. Potential long-term benefits to women and children’s health and development could come from making comprehensive maternity protection available and accessible to all women.

**Supplementary Information:**

The online version contains supplementary material available at 10.1186/s12884-022-04944-0.

## Background

Globally, 61.2% of employed people work informally, and in certain regions of the world, such as Africa, 85.8% of employment is informal [1]. Informal employment refers to a range of working relationships that generally do not have legal or social protection, where employers do not comply with national labour legislation, do not pay income tax, and workers or employees are not entitled to employment benefits like paid leave [2]. Informal employment can take place inside and outside the informal sector. The informal sector refers to usually small organisations that are not registered, have a low level of organisation, elude government regulatory requirements and are often managed from informal arrangements such as households and street pavements [3]. Non-standard employment relationships are described by the International Labour Organization (ILO) as temporary employment (fixed-term contracts including project-based contracts, seasonal work, casual work and daily work), part-time and on-call work, multi-party employment (also known as temporary agency work or subcontracted labour) and disguised employment or dependent self-employment (such as platform work) [4]. Those working in positions of non-standard employment will hereon be referred to as non-standard workers.

Domestic workers have been described as informal wage workers, where they are hired generally without social protection and by informal enterprises [2]. Domestic workers work in other people’s households and sometimes live at their workplace. Some domestic workers work for one employer full-time; others work for different employers on different days of the week. In South Africa (SA), some domestic workers are employed through a platform (e.g., SweepSouth) which presents a complicated employment relationship. In SA, attempts have also been made to regulate and formalise the domestic work sector, for example, through the establishment of the Sectoral Determination for the Domestic Work sector in 2002 [5]. Therefore, domestic work is heterogenous with different levels of formality.

Women in positions of non-standard employment are vulnerable to receiving inadequate maternity protection due to informal employment arrangements [6]. Comprehensive maternity protection includes health protection at the workplace, a period of maternity leave, cash payments and medical benefits while on maternity leave, job security (employment protection), non-discrimination, daily breastfeeding breaks and childcare support [7]. Access to all components of maternity protection is needed to successfully combine work and breastfeeding, yet in research and programme implementation, the focus appears to mainly be on paid maternity leave, breastfeeding breaks, and childcare. Information on the accessibility of maternity benefits for non-standard workers is limited [8] and the full package of maternity protection may seem unrealistic. All working women who are pregnant or breastfeeding, including those in atypical forms of dependent work, should be able to access comprehensive maternity protection, and this would provide women and children access to health and social protection [9].

In SA, women in non-standard employment make up 30.1% of the female workforce [10]. The informal sector refers to organisations that employ less than five people and do not deduct income tax from wages [11]. Domestic workers working in private households are excluded from the Statistics SA definition of the informal sector [12]. Most domestic workers (94.5%) in SA are women [10]. Maternity leave and most general employment protection is regulated through the Basic Conditions of Employment Act of the National Department of Employment and Labour, formerly the National Department of Labour [13]. The Unemployment Insurance Fund (UIF) enables access to payment of 66% of a woman’s previous earnings while on maternity leave. In 2021, only 59% of all employed women could confirm that they contribute to the UIF [10] while in 2019 only 20% of domestic workers reported being registered for the UIF [14]. Many women, particularly those working outside of formal employment may be ineligible for UIF maternity benefits (such as domestic workers who are eligible but not registered by their employers). Eligible women, based on a means test, can apply for social assistance through the national social grant scheme once the child has been born. The maternity protection landscape in SA is complicated and inadequately understood. A recent policy analysis showed that maternity protection is dispersed throughout different legislative and policy documents located in different sectors [15]. Although most of the ILO minimum requirements for maternity protection are present in SA policy, implementation is unclear and inconsistent for women in non-standard employment. Building on a recent policy analysis which described the broad maternity protection policy environment in SA [15], this study aimed to describe maternity protection available to women in positions of non-standard employment in SA, using domestic workers as a case study.

## Methods

### Study design

**Fig. 1:**
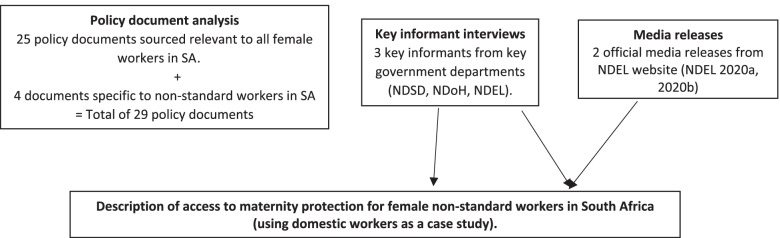
Flow chart describing data collection methods. NDSD = National Department of Social Development; NDEL = National Department of Employment and Labour, NDoH = National Department of Health; SA = South Africa

A qualitative descriptive study design was used to explore and illustrate the current maternity protection benefits available to women in positions of non-standard employment in SA. As is typical of public health policy analysis, data collection techniques included a combination of document analysis and key informant interviews [16] (Fig. 1) together with synthesis from published literature.

### Setting and relevant context

South Africa is a middle income country with high rates of poverty, inequality and unemployment [17]. In 2019, approximately 18 million South Africans (almost one-third of the population) were receiving some sort of social assistance from government [18]. Figures from 2021 show that 69.9% of working women were employed in the formal sector, with the remainder working in the informal sector (13.8%), private households (12.8%) and agriculture (3.5%) [17]. Of all working women, 13% work as domestic workers [17].

### Sample

#### Document analysis

During 2020, current national policy documents containing any provisions relevant to maternity protection in SA were sourced by the first author (CPK), using ILO guidance on the types of documents to search [19] together with evaluating previous reviews on similar topics [20–22]. In this research, policy documents refer to any policy tool used to implement policy, including the Constitution, legislation and regulations, national policies, and national guidelines (e.g., codes of good practice, national guidelines, etc.). The ILO describes that maternity protection is usually located in labour, social security, anti-discrimination, and health policy and legislation [23]. Documents were sourced by searching the websites of respective national government departments and included if they contained at least one provision of maternity protection. Documents were categorised as: legislation (legally enforceable), policy (enforceable by the department responsible) or guidelines (non-legally binding recommendations). Documents published from 1994 (following the establishment of a democratic government in SA) until September 2021 (most recent) were included. A total of 29 national level policy documents were identified that contained provisions relevant to maternity protection for non-standard workers.

#### Key informant interviews

Key informants were purposefully selected based on their position to influence national maternity protection policy in SA in order to gather information that could assist in understanding the context and process of national policy development for maternity protection. An analysis of maternity protection for all women revealed that the key government departments identified to be involved in maternity protection provision are the National Departments of Employment and Labour, Health, and Social Development [15]. Therefore, a key informant was selected, at the level of Assistant or Deputy Director, based on their experience of working in each of these departments.

### Data collection

#### Document analysis

Documents were identified by CPK between August 2020 and September 2021. The document analysis is described in detail in the recent policy analysis on maternity protection for all working women in SA compared to global recommendations [15]. Information was extracted from documents and entered into a Microsoft Excel spreadsheet according to title, date, author, publisher, sector, document type, purpose of document, target audience and the component/s of maternity protection addressed by the document. Existing published reviews on maternity protection policies were also sourced to compare this research to existing interpretations of maternity protection policy in SA in the context of ILO recommendations. Two official media releases were also used, as they were referred to by a key informant and deemed relevant.

#### Key informant interviews

To supplement the document review and analysis, and to provide context to the content of the policy documents review, individual in-depth interviews (IDIs) were held with three key informants during October and November 2020. The IDIs explored national maternity protection policy development and implementation. Therefore, an interview guide was developed that aimed to gain insights into policy content and implementation (see Additional file 1). The IDIs were conducted using a virtual platform of the interviewee’s choice. Interviews were on average 45 min and conducted in English by CPK. IDIs were audio-recorded and transcribed by CPK.

### Data analysis

In this study, we used the “READ approach” to analyse maternity protection policy documents, which includes to *“(1) ready your materials, (2) extract data, (3) analyse data and (4) distil your findings”* ([24], p1424). All documents were assigned a label. Relevant content was extracted and captured into a Microsoft Excel spreadsheet. Policy content was analysed by identifying text that referred to any ILO defined component of maternity protection; the text was then coded manually according to which component of maternity protection it referred to. Policy content specific to non-standard workers was extracted and described. The IDIs were analysed manually by CPK, who read and re-read transcripts, allocated codes to similar groups of information and developed overarching themes linked to the codes. The data extracted from published policy documents and information obtained from the interviews were triangulated by interpreting the content of policy documents within the context of the responses from key stakeholders. A thematic analysis approach was used for evaluation of policy content and for the analysis of the IDIs [25].

### Ethics

All documents describing and analysing the policy content were publicly accessible. Participants gave verbal informed consent for the individual IDIs and agreed to the interviews being audio-recorded. All interview data was stored electronically and securely by CPK. Participants’ confidentiality was maintained by removing personal information and names linked to individuals’ insights from the transcribed data in any reporting of the results. Privacy, confidentiality, and anonymity was ensured. Ethical approval was obtained from the University of the Western Cape’s Senate Research Committee and Ethics Committee [Reference Number: BM20/5/7].

## Results

**Fig. 2:**
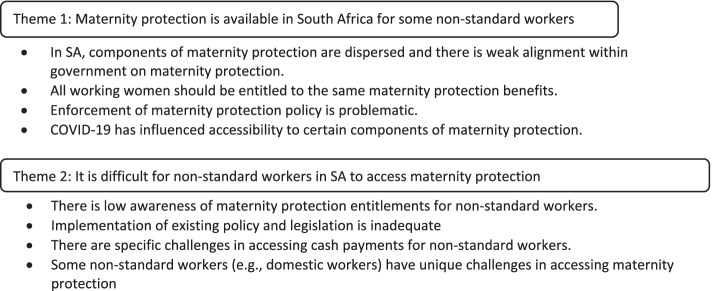
Themes and sub-themes emerging about availability and accessibility to maternity protection in South Africa

The 29 policy and legislative documents from which information on maternity protection in SA was obtained are listed in Table 1. The components of maternity protection and documents where they are located are summarised in Table 2. The information obtained from the policy documents, three key informant interviews and two media releases (Fig. 1) are used to describe availability and accessibility of maternity protection for non-standard workers in SA, using domestic workers as a case study. The three major themes and sub-themes that emerged from the analysis of documents and interviews are illustrated in Fig. 2.

**Table 1:** Policy and legislative documents relevant to maternity protection in South Africa

**Constitution** Department of Justice: Constitution of the Republic of South Africa (1996)
**Legislation**
Department of Labour: Labour Relations Act (1995) • Amendment No. 12 of 2002 • Amendment No. 6 of 2014
Department of Labour: Basic Conditions of Employment Act (1997) • Amendment No. 11 of 2002 • Sectoral Determination 7: Domestic Worker Sector of 2002 • Sectoral Determination 13: Farm Worker Sector of 2006 • Amendment No. 20 of 2013
Department of Labour: Employment Equity Act (1998) • Amendment No. 47 of 2013
Department of Labour: Promotion of Equality & Unfair Discrimination Act (2000)
Department of Labour: Unemployment Insurance Act (2001) • Amendment No. 10 of 2016 • Amendment Regulations 2018
Department of Labour: Unemployment Insurance Contributions Act (2002)
Labour Laws Amendment Bill 2017
**Policy**
Department of Health: Infant & Young Child Feeding Policy (2013)
Department of Public Service & Administration: Determination and Directive on Leave of Absence Policy (2015)
**Guideline**
Department of Labour: Code of Good Practice on the Protection of Employees during Pregnancy and after the Birth of the Child (1998)
Department of Labour: Code of Good Practice on the Arrangement of Working Time (1998)
Department of Labour: Code of Good Practice on the Integration of Employment Equity into Human Resource Policies and Practices (2005)
Department of Health: Tshwane Declaration of Support for Breastfeeding in South Africa (2011)
Department of the Presidency: National Development Plan (2012)
Congress of South African Trade Unions (COSATU): Maternity Protection Position Paper (2016)
Department of Health: Nutrition Guidelines for Early Childhood Development Centres (2017)
Department of Health: Supporting Breastfeeding in The Workplace Booklet (2018)
Socio-economic Rights Institute (SERI): Domestic Workers’ Rights: A Legal and Practical Guide (2018)
Project 143: Discussion Paper 153 on Maternity and Parental Benefits for Self-employed Workers in the Informal Economy (2021)

**Table 2:** Provisions of maternity protection and their location in policy or legislative documents

**Maternity leave**
Basic Conditions of Employment Act (1997) Code of Good Practice on the Integration of Employment Equity into Human Resource Policies and Practices (2005) Tshwane Declaration of Support for Breastfeeding in South Africa (2011) Leave of Absence Policy (2015) Maternity Protection Position Paper (2016)
**Cash payments and medical benefits**
Basic Conditions of Employment Act (1997) Employment Equity Act (1998) Unemployment Insurance Act (2001) Unemployment Insurance Contributions Act (2002) Code of Good Practice on the Protection of Employees during Pregnancy and after the Birth of the Child (1998) Code of Good Practice on the Integration of Employment Equity into Human Resource Policies and Practices (2005) National Development Plan (2012) Leave of Absence Policy (2015) Maternity Protection Position Paper (2016)
**Health protection**
Basic Conditions of Employment Act (1997) Code of Good Practice on the Protection of Employees during Pregnancy and after the Birth of the Child (1998) Code of Good Practice on the Integration of Employment Equity into Human Resource Policies and Practices (2005) National Development Plan (2012) Maternity Protection Position Paper (2016)
**Employment protection (job security)**
Labour Relations Act (1995) Employment Equity Act (1998) Maternity Protection Position Paper (2016)
**Non-discrimination**
Constitution (1996) Labour Relations Act (1995) Basic Conditions of Employment Act (1997) Employment Equity Act (1998) Code of Good Practice on the Protection of Employees during Pregnancy and after the Birth of the Child (1998) Promotion of Equality & Unfair Discrimination Act (2000) Code of Good Practice on the Integration of Employment Equity into Human Resource Policies and Practices (2005) Maternity Protection Position Paper (2016)
**Breastfeeding breaks**
Code of Good Practice on the Protection of Employees during Pregnancy and after the Birth of the Child (1998) Code of Good Practice on the Arrangement of Working Time (1998) Tshwane Declaration of Support for Breastfeeding in South Africa (2011) Infant & Young Child Feeding Policy (2013) Supporting Breastfeeding in The Workplace Booklet (2018) Maternity Protection Position Paper (2016)
**Childcare**
Code of Good Practice on the Arrangement of Working Time (1998) Code of Good Practice on the Integration of Employment Equity into Human Resource Policies and Practices (2005) Maternity Protection Position Paper (2016) Nutrition Guidelines for Early Childhood Development Centres (2017)

### Theme 1: Maternity protection is available in South Africa for some non-standard workers

#### In South Africa, components of maternity protection are dispersed and there is weak alignment within government on maternity protection

Most components of maternity protection (maternity leave, medical benefits, health protection at the workplace, employment protection, non-discrimination, breastfeeding breaks, and childcare) are described in 29 national policy and legislative documents; and the various components of maternity protection are dispersed across these documents (Table 2). The National Department of Employment and Labour has main legislative responsibility for maternity protection, but other departments and organisations also have policies and guidelines containing recommendations for implementing the different provisions of maternity protection.

This separation of maternity protection across different policies and departments is confusing. Furthermore, fragility in coordination between government departments was described as a barrier to effective implementation of maternity protection. One key informant described how poor communication between two key government departments is a potential barrier to policy implementation:

“…because even from where we [Department of Health] are sitting, we are not sure as to who is directly responsible within the Department of Labour, with regards to these matters … so I'm not sure … what is happening, which platforms are these discussions being held? Is there a draft something that is available? That maybe was issued out for public comment. I have no idea…” (Key Informant 2)

The above response reflects an uncertainty from an individual who has influence over policy due to their level of employment in government. The response also illustrates a siloed approach in government to policy development with weak alignment between government departments.

Key informants had the view that at national government level where priorities are determined, maternity protection is not always assigned priority. This was voiced as being because the impacts of maternity protection policy implementation are not immediately visible. This can influence the policy and legislative process, as one key informant described that priority is allocated (especially by politicians) to actions that can be achieved within a short timeframe:

“…unfortunately, the problem with our politicians, and I know, it’s not only in South Africa, politicians… want[s] short term… when we look at this work, it’s a future investment, you see, investing in human capital…” (Key Informant 1)

#### All working women should be entitled to the same maternity protection benefits

In current maternity protection policy and legislation, certain entitlements are defined as being applicable to all workers, namely maternity leave, medical benefits, employment protection (job security), non-discrimination, breastfeeding breaks, and support with childcare responsibilities. However, the availability of cash payments while on maternity leave (enabled through social insurance) is defined differently in legislation for those working less than 24 h per month for an employer. Social insurance is not available to certain groups of non-standard workers in SA, such as self-employed workers in the informal economy [26]. The perception of the key informants was that all women should receive equal maternity protection through current legislation and policy, however there are differences in how this protection can be accessed:

“… the labour laws should protect everyone, equally. And there’s huge gaps or discrepancies or this inequality, I think, when you look at [it] from a social perspective … especially the more informal sector, like as it may be the case of domestic workers. They are excluded from this kind of benefits … paid maternity leave is not guaranteed, it’s something that is voluntary.” (Key Informant 2)

“Well, I don’t think it should be different. I think it should be the same. The law protects us equally, it’s now just that the only difference is that as a domestic worker, I’m employed by an individual. But then personally, I feel that they need to get equal protection, much as the law covers us equally, but in practice, its different, the implementation in practice is different.” (Key Informant 3)

The responses from the key informants reveal differences in interpretation of how policies and legislation apply to different sub-groups of non-standard workers (e.g., domestic workers).

Furthermore, certain groups are completely excluded from specific components of maternity protection. Project 143’s Discussion Paper 153 on Maternity and Parental Benefits for Self-employed Workers in the Informal Economy describes how various groups of non-standard workers are currently excluded from social insurance in SA, meaning that they are not eligible to claim cash payments while on maternity leave [26]. This group of workers includes women informal workers, waste pickers, farm workers, taxi industry workers, street vendors, home-based workers, caterers and decorators, fishers, freelance artists, and informal childcare workers. Therefore, comprehensive maternity protection is not available to all non-standard workers.

#### Enforcement of maternity protection policy is problematic

Implementation of laws, policies and guidelines was described as weak because enforcement capacity is insufficient. Challenges described by key informants were practical logistics that can prevent adequate enforcement of maternity protection policy and workers’ fears of the consequences of reporting their employers. Labour laws are supposed to be monitored and enforced by labour inspectors. One key informant reported that there are not enough labour inspectors in SA. This was confirmed by a media statement in 2020 shared by the key informant, where the Director-General for the National Department of Employment and Labour stated that while the Department has over 1 500 inspectors, this is insufficient for the 1.8 million employers registered on the Unemployment Insurance Fund's database [27].

It was also described that monitoring of maternity protection policy is a challenge and is done in a reactive rather than proactive manner. Workers may also be hesitant to report employers not complying with legislation due to worries about future job security:

“…so I think those are the big gaps, even the recommended four months of maternity leave, you know, I’m not sure if Labour is really monitoring this kind of things, so they wait for people to come in and perhaps complain, to say, my employer doesn't want to register me, and you know that there is some risk that you know people might not even come forth to say I'm not registered, my employer doesn’t want to do that, because of fear of – losing their jobs…” (Key Informant 2)

The key informant from labour also stated that labour inspectors do not have enough strength and power to enforce legislation:

“… that issue of them maybe being able to issue fines and all those, I mean if the legislation is maybe amended to empower the inspectors to be able to, maybe issue, even spot-on fines and all that, but… if that was possible, I think that would actually maybe improve things a bit, especially for informal workers.” (Key Informant 3)

However, when questioned about this, the key informant indicated it would be highly unlikely for labour inspectors to have their authority increased to that of issuing fines. Therefore, the current enforcement mechanisms for not complying with labour legislation appear to be inadequate.

#### COVID-19 has influenced accessibility to certain components of maternity protection

It was acknowledged by one key informant that when national policy is being finalised, there are contextual factors that may influence policy priority. At the time of interview, government’s response to the COVID-19 pandemic, together with the enactment of a national basic income grant, were listed as policy priorities where time and resources may be re-directed:

“…and also, there’s competing priorities, as you would know, now, there’s—with COVID, the basic income grant…”. (Key Informant 1)

The COVID-19 pandemic has been a significant competing priority for government and from March 2020, this impacted the functioning of the National Department of Employment and Labour (NDEL). Financial resources were used from the UIF (the fund where maternity leave payments are paid from) to make payments to individuals who could not work during times of national lockdown. By October 2020, the NDEL reported that the UIF had paid out more than R51 billion in Covid-19 Temporary Employer-Employee Relief Scheme payments of R350 per person per month for those unable to work due to lockdown regulations [28]. Therefore, the COVID-19 pandemic resulted in additional social assistance being made available to workers, which both depleted finances from the social insurance fund but also diverted human resources needed to administer social assistance.

### Theme 2: It is difficult for non-standard workers in South Africa to access maternity protection

#### There is low awareness of maternity protection entitlements for non-standard workers

Key informants explained that there is lack of awareness (among employers and workers) regarding maternity protection entitlements, particularly for non-standard workers:

“I think one of the gaps is lack of knowledge, perhaps if people know of these laws, and what can be done, what can’t be done, perhaps some of them can be better educated and know more and be able to assist us in complying to implementation of this laws… If people are not aware of something, they will not demand for it.” (Key Informant 2)

One key informant described that it is the labour department’s responsibility to create advocacy around maternity protection that all workers should be entitled to:

“…they do have… awareness raising where they educate domestic workers to say… come to the Department and find out if you are registered, so that if you are not registered, we can then follow up on your behalf.” (Key Informant 3)

A challenge related to advocacy by labour inspectors is that work performance targets are measured by inspections done and advocacy is therefore deprioritized:

“… as Labour Inspectors, how they operate… they have targets they have to meet in terms of … proactive inspections, therefore, you will find that most of the time that is where the focus is because that is where they will be assessed and asked is that where you are performing or not? So now, in terms of other campaigns and other sorts of work they need to do, it now becomes less of a priority.” (Key Informant 3)

It appears that there is low priority for creating awareness and increasing knowledge on maternity protection entitlements for non-standard workers. This makes it challenging for workers to access these protections.

#### Inadequate implementation of existing policy and legislation

In SA, certain categories of workers are protected by sectoral determinations, an additional legal measure intended to protect certain sectors, established by the labour department [13]. Sectoral determinations prescribe minimum rates of remuneration and certain conditions of employment in specific sectors (e.g., minimum standards for housing and sanitation if workers live on employers’ premises, regulation of work-related allowances, regulation of benefits such as pension, medical aid, leave, unemployment funds, etc.) [13]. Sectoral Determination 7 was established for Domestic Workers [5] and Sectoral Determination 13 for Farm Workers [29]. Even though sectoral determinations contain provisions for employment conditions, those working less than 24 h per month for an employer are effectively only protected by the minimum wages standards of the sectoral determinations [30]. Provisions related to maternity leave in the sectoral determinations simply state that women in these sectors should be able to access the same benefits as all workers. The Sectoral Determination 7 for Domestic Workers states that from 2003, domestic workers will be entitled to contribute to and claim cash payments from the UIF through the Unemployment Insurance Act of 2001 [5]. Cash payments and breastfeeding breaks are not described or mentioned in these sectoral determinations. These sectoral determinations do not actually provide much more protection in practice, and simply repeat basic maternity protection provisions described in other labour laws as being applicable to domestic workers and farm workers, without any regard to the heterogenous nature of employment in these sectors. Therefore, the existence of these sectoral determinations for some groups of non-standard workers is insufficient, since they are not being adequately monitored and enforced for implementation.

While policy is usually developed at a national level, implementation takes place at the provincial (i.e., sub-national) level. It was described that maternity protection policy may be less well implemented at the provincial level:

“… when you go to the provincial level, that’s where you see … disjuncture between policy development, and implementation…” (Key Informant 1)

Therefore, even though there is national policy and legislation for most components of maternity protection, some of which applies to non-standard workers, this does not guarantee its implementation. Therefore, many women working informally remain unprotected. One key informant recommended that simply implementing existing maternity protection for all would be beneficial to non-standard workers:

“I think so far, the protection that is currently available in the law, if enforced, it would go a long way.” (Key Informant 3)

The following section provides further examples of how the cash benefit component of current maternity protection legislation is inadequately implemented.

#### Limited cash payments are available to non-standard workers while on maternity leave

Non-standard workers have difficulty accessing cash payments while on maternity leave. Access to social insurance (and therefore cash payments while on maternity leave) is complex and is different for certain non-standard workers. Only those working at least 24 h per month (average of 6 h per week) for an employer can register with the UIF and participate in the social insurance scheme [31, 32]. Those working less than 24 h per month are considered part-time workers [30]. Social insurance provides temporary relief and the amount received is related to how long a worker has been contributing to the fund. Certain non-standard workers (for example, domestic workers) may work for multiple employers in a month, sometimes working for different employers on different days of the week and may not be working more than 24 h for a single employer in a month. These workers would be excluded from participation in the social insurance programme in SA. To be able to claim social insurance while on maternity leave (from the UIF), employers need to register their workers and both employers and workers need to contribute 1% of monthly earnings to the UIF.

Key informants described that not all workers, especially those working in informal sectors and domestic workers, are registered with the social insurance scheme (the UIF):

“…there’s a gap within these people who work within informal sectors, wherein sometimes you are not even registered to be employed.” (Key Informant 2)

“…and now that is a challenge, because with the domestic workers, most of them are not registered.” (Key Informant 3)

For women unable to access social insurance, the only access to cash payments while on maternity leave may be in the form of social assistance. In SA, after the birth of a baby, women can apply for social assistance, in the form of the monthly Child Support Grant which is available monthly to caregivers of children under 18 years of age earning insufficient income, as determined by “means test” criteria [33]. In 2022/23, the value of the Child Support Grant was R480 (30 US dollars) per month for each child under 18 years [34]. This is much less than a monthly salary calculated at the National Minimum Wage Rate (R3 710) [35]. However, the CSG may be the only financial assistance that women working informally can access after delivering a baby while on maternity leave. Since cash payments while on maternity leave are a component of maternity protection that may be difficult to access, this was probed further. One key informant described that there are different opinions regarding the value of providing social assistance with not all stakeholders agreeing on its priority:

“…particularly social assistance, is a very contested space, you see even internally – when I say internally, I mean within the Department, of Social Development, you still have people who are not seeing a value in this, you see, so it’s very difficult to tell.” (Key Informant 1)

It was also emphasized that social protection in SA is fragmented, and one key informant recommended that social assistance be linked to other services, departments, and sectors, implying that improved coordination of services is required. Although inadequate to replace income, the Child Support Grant is a more certain mechanism whereby women receiving unpaid maternity leave can obtain social assistance and women receive this assistance until the child is 18 years. One key informant described however, that even this route of obtaining cash payments by non-standard workers is inadequately implemented:

“…we’ve been having a problem of… lots of eligible children, particularly aged zero to four, who are not accessing the [child support] grant, we’ve got research that tells us that around 3 million children, who are eligible, are not receiving… [the child support grant]” (Key Informant 1)

Therefore, social assistance is an insufficient form of cash payments while on maternity leave and provides another example of challenges in implementing existing social policy.

#### Some non-standard workers (e.g., domestic workers) have unique challenges in accessing maternity protection

Domestic workers are employed by individuals and a domestic worker’s place of work is a private household which is difficult to monitor. This can result in inconsistent implementation of maternity protection legislation that depends on the knowledge and practices of the individual employer. One key informant described how many employment benefits for domestic workers are at the employer’s discretion:

“…when it comes to the whole thing about the additional benefits, like you know, being absent because you’re ill, it depends, you’re at the mercy of your employer, who will… feel sympathetic and empathetic to say oh… this domestic worker is so good, maybe I should also return the favour and give them time off…” (Key Informant 2)

When asked about the maternity protections that are supposed to be guaranteed according to SA legislation, one key informant responded that certain provisions are simply unavailable to domestic workers:

“…some people know that if you don't pitch for work whether you're pregnant or not… it's a deduction, you don’t get full pay. Those kind of things, breastfeeding breaks are, I mean, it’s even out of question…” (Key Informant 2)

Regarding health protection at the workplace, women in positions of non-standard employment (e.g., domestic workers, agricultural workers, and informal vendors) may more commonly be in situations where they are required to do physically demanding work, inappropriate for a woman in the later stages of pregnancy or soon after the delivery of a child.

A challenge described as unique to domestic workers is that their workplaces are households, which are private spaces and therefore difficult to access and monitor:

“Domestic work happen[s] in a private household, so there's a challenge of access to a private household, so that automatically becomes a challenge for the department [to] even monitor … by the companies where they can just come in unannounced and take the books and do a spot check, so unfortunately it is a challenge in that regard.” (Key Informant 3)

Therefore, certain characteristics of the non-standard employment relationship mean that the enforcement of maternity protection for domestic workers is especially challenging.

## Discussion

This study aimed to describe the components of maternity protection available and accessible to non-standard workers in SA, investigating domestic workers as a case study. According to policy and legislation, all working women should be eligible to mostly the same maternity protection, but characteristics of non-standard employment relationships make it difficult for some groups to access certain components of maternity protection and accompanying benefits. Since women in the informal economy make up a significant proportion of the workforce, especially in Africa, it is important to consider their labour-related rights. We have described which components of maternity protection may be difficult to access and the factors influencing non-standard workers’ access to these. An accurate and up-to-date description of maternity protection entitlements for non-standard workers in SA was previously not available and is needed to advocate for and improve women’s access to these entitlements. The combined methods used (document analysis and key informant IDIs) allowed for information relevant to policy content to be extracted from documents, and for key informants to describe and interpret selected provisions of documents in more depth. The results from this research show that there is confusion regarding maternity protection entitlements of non-standard workers. This is probably because maternity protection is dispersed across various policy locations [15] and is difficult to define for all female workers. The entitlements for non-standard workers are particularly unclear due to heterogeneous working conditions and varied employment relationships. Certain components of maternity protection are unavailable or inaccessible for certain groups of non-standard workers. Improved access to maternity protection could improve maternal health and contribute to breastfeeding support for working women [36]. This in turn has potential long-term benefits for women’s and children’s health and development. Expanded social protection could contribute to reducing poverty and improving livelihoods for women and their families [37].

An important component of maternity protection is access to cash payments while on maternity leave, especially in countries with high rates of poverty and low incomes, like SA [38]. If a woman can’t access cash payments while on maternity leave, she may need to return to work early, and therefore may not make use of the full maternity leave benefit available to her. This can have implications on other health and childcare practices, such as breastfeeding [36]. The results from the document analysis and key informant interviews clearly demonstrate that current routes of access to cash payments while on maternity leave for non-standard workers are problematic to navigate. In SA, some employers, usually those in formal employment, facilitate maternity benefit claims from the UIF on behalf of their employees, and some top up that amount to ensure that women receive their full salary while on maternity leave. For women not contributing to the UIF, their employers may voluntarily pay their salary (in part or full) while on maternity leave. However, this is not guaranteed especially for women without contracts. A woman may only find out that she and the employer have not been contributing to the UIF when she goes on maternity leave and struggles to claim cash payments from the fund. Women who can’t access paid maternity leave or social insurance need to rely on state social assistance (the child support grant). However, this is less than minimum incomes and many women are unable to access the child support grant soon after childbirth [39] with some only accessing this support once they have returned to work after maternity leave. Most non-standard workers earn unstable and low incomes and are therefore unable to accumulate savings for their maternity leave period. Women need to receive sufficient income while on maternity leave. There have been calls for social assistance to start during pregnancy in SA to improve maternal and child health outcomes [40, 41]. While it could be recommended that large employers provide mandatory payment to employees while on maternity leave, for non-standard employment relationships this may not be feasible. SA should ratify the ILO’s Maternity Protection Convention 183 and maternity leave, paid at 100% of previous earnings, should be available to all women. Current legislation creates an unconscionable risk to women who may lose their income for the months they are on maternity leave.

The results from this research demonstrate there are notable silences regarding certain components of maternity protection policy for women in non-standard employment relationships. Therefore, non-standard workers are particularly vulnerable to inadequate maternity protection. South Africa is used as an example globally, of how social protection has been extended to people dependent on the informal economy, by the expansion of the UIF (social insurance) to include domestic workers [3]. This is problematic because firstly, even though domestic workers are legally protected in SA, through the Sectoral Determination for Domestic Work established 20 years ago, most domestic workers are not able to access social insurance. In 2019 only 20% of domestic workers reported being registered for the UIF [14]. Secondly, there are many groups of non-standard workers that are still currently excluded from social insurance in SA. Current legislation should be strengthened and amended so that social insurance is available to all categories of non-standard workers. Project 143’s Discussion Paper 153 proposes draft legislation, via the recommendation of a Draft Bill: Social Assistance, Employment and Labour Laws General Amendment Bill to extend maternity and parental benefits to self-employed workers in the informal economy [26]. An ILO report recommended that expanding social insurance coverage to non-standard workers would assist to ensure health and well-being of more women and their children [42]. There have also been suggestions that a combination of formal social protection systems together with acknowledgement of the role of informal or traditional support, such as families and communities assisting with unpaid childcare, needs to be better recognised [3].

Researchers in Asia have acknowledged similar challenges to SA. In some Asian countries, most employed women work in the informal economy and are excluded from social security programmes that provide cash payments to women on maternity leave. The annual financing needs to provide non-contributory maternity cash transfers to women on maternity leave in the informal economy has recently been calculated for the Philippines [43]. These calculations have been shown to be financially feasible for the Philippines since the requirement would be less than 0.1% of the country’s annual gross domestic product. This would be less than the cost of not breastfeeding which is estimated to be 0.7% of the Gross Domestic Product. The researchers therefore recommend that the provision of cash transfers to women on maternity leave in the informal economy would be a good social investment [43]. This conclusion could also apply to other low-and-middle-income countries (LMICs), including SA.

The results from this research also show that implementation of existing legislation is suboptimal and that there is weak alignment across government departments. Government, specifically the national Department of Employment and Labour, needs to ensure that the efficiency and accessibility of current social protection mechanisms (e.g., the UIF) are improved. Social protection, including maternity protection, which is currently fragmented, needs to be unified in South Africa. In August 2021 a Green Paper on Comprehensive Social Security and Retirement Reform for SA was published. It described that maternity and pregnancy support is being considered separately [44]. The Green Paper recommended a comprehensive coherent system for social protection in SA including the establishment of a national social security fund but was unfortunately withdrawn soon after publication. A more coherent, inter-sectoral approach to social protection is needed.

Research conducted with domestic workers living in Gauteng, an urban populous province in SA, the majority of whom were migrant workers, revealed domestic workers experiencing many basic human rights violations (e.g., physical and/or verbal abuse) and risk for domestic workers being dismissed if they are pregnant or upon return from maternity leave [45]. General labour rights violations (such as not having a written contract, not contributing to social insurance or being paid below the minimum wage) have also been documented for other groups of non-standard workers in SA, such as farmworkers [46]. Research on availability and accessibility of comprehensive maternity protection for all groups of non-standard workers especially in LMIC is currently limited [8] and therefore required. There is a need for advocacy campaigns and improved awareness of both employers and workers regarding the maternity protection rights that all female workers are entitled to according to SA legislation.

There are similar challenges to ensuring that maternity protection is available and accessible to non-standard workers in other regions with high numbers of LMICs. Although the overall trends in Southern and Eastern Africa are for longer and better paid maternity leave funded by social insurance, there are still many countries that rely on employers to fulfil maternity income protection obligations, and even in countries with established social insurance systems, non-standard workers are often inadequately protected [47]. Similarly, in Latin America and the Caribbean, social protection mostly benefits those working formally, even though over half of workers are in the informal sector and in some countries in the region, the financial of maternity leave depends on the employer [48]. It is not a new recommendation that labour laws be revised to include informal workers and provide social protection for breastfeeding women with low incomes [49] and this is not a problem that is unique to SA. Therefore, lessons learned from the SA context could be used and applied to other LMICs with high rates of non-standard employment and similar challenges in accessing maternity protection.

### Limitations

Despite efforts to address researcher bias and reflexivity, it is possible that some bias remains. While the document search was extensive, it is possible that some documents were not included. Although purposively selected as key opinion leaders on the topic, the small number of key informants interviewed and that they are only representatives from national government departments is a limitation. The use of purposive sampling may have led to selection bias. There are many migrant workers often from neighbouring countries that take up positions of non-standard employment (including domestic work) in SA and this category of non-standard worker has not been considered in this manuscript.

## Conclusions

In SA, currently all components of maternity protection are not available and accessible to non-standard workers. The heterogeneity of non-standard employment makes it even more challenging for many women to access maternity protection. However, there are policy amendments that could be made and improvements to policy implementation that would improve non-standard workers’ access to maternity protection. Lessons learned from the SA context could be applied to other LMICs where non-standard employment is common and similar challenges to access maternity protection are experienced. We should not lose sight of the potential long-term benefits to women and children’s health and development that would come from making comprehensive maternity protection available and accessible to all women.

## Supplementary Information

**Additional file 1.**

## Data Availability

The documents included for analysis are all publicly available. The datasets generated and/or analysed during the current study are not publicly available to protect participant confidentiality but are available in an anonymised form from the corresponding author on reasonable request.

## Abbreviations

IDI: In-depth interview
ILO: International Labour Organization
LMICs: Low-and-middle-income countries
SA: South Africa
UIF: Unemployment Insurance Fund
