# Supplementary material for: Maternity protection for female non-standard workers in South Africa: the case of domestic workers
Source: BMC Pregnancy Childbirth. 2022 Aug 22;22:657. doi: 10.1186/s12884-022-04944-0 (PMC9394052; doi:10.1186/s12884-022-04944-0)
Supplement: Supplementary file 1 — Additional file 1. [file 12884_2022_4944_MOESM1_ESM.docx]

**Interview guide for Individual Semi-structured Interviews with Key Informants**

**Purpose of interview:** to understand the process, actors, context, and power with regard to maternity protection policy in South Africa (with a focus on non-standard employees).

Date: _________________________

**Key Informant Description (demographic variables)**

Sector: _______________

Type / Name of Organization: _______________

Department Within Organization: _______________

Position Within Organization: _______________

Sex: ________________

Race: ________________

Hi, my name is XXXX. Thank you for taking the time to speak to me. I am doing my PhD in through the University of XXXX. The overall aim of my PhD research is “To examine current maternity health and economic protection benefits available and accessible to non-standard employee domestic workers, in the Western Cape, to improve understanding of potential implications for breastfeeding practices”.

*You can play this by ear depending on who you interview, unlike in academic writing, you may need to put this a bit more colloquially, for e.g.*

“In my PhD research I am aim to examine what existing maternity health and economic benefits are available to workers such as domestic workers in the Western Cape. I am particularly interested in knowing more about whether they are able to access the benefits that do exist and if has any effect on whether they are able to continue breast feeding their babies if they go back to work”. *Or something similar to this.*

As part of my research, I would like to ask you a few questions. The reason I have asked to speak to you is that XXXX recommended that you are in a position at the National Department of XXXX, to be able to answer a few questions regarding maternity protection benefits in South Africa. Your knowledge and experience can help me to understand a bit about how policy and legislation regarding maternity protection is developed in South Africa – who or which sectors or departments have influence and responsibility for maternity protection and how the current policy/legislation has come to be the way it is (i.e., the context within which legislation has been developed and the process of the development of legislation).

1. Firstly, in my research, I am using the term “maternity protection benefits”. What is your understanding of what comprehensive “maternity protection benefits” refer to? What benefits do you think that women should be able to access in South Africa, when they are working and then have a baby? (*Prompt/Probe: Can you list the benefits that women should be able to receive when they are pregnant and following the birth of a child?*)
2. Can you tell me about any specific policies or legislation (laws) you are aware of that exist to ensure that women are able to access maternity protection?
3. Do you think that your department has a role to play in ensuring that women can access maternity protection benefits and if so, what is that role?
4. Which departments or sectors or even individuals do you think have the most power, influence, and responsibility when it comes to ensuring that women can access maternity protection benefits? Could you tell me about the reasons for this?
5. Can you tell me a bit about what you know about the process with which maternity protection legislation has been developed over the years in South Africa?
6. Are you aware of the position of your department regarding the ratification of the International Labour Organization’s Maternity Protection Convention? At a meeting I attended in 2018, it was stated that South Africa was aiming to ratify the convention by 2020. Do you know if this is still the goal or aim? If yes, can you tell me about what you know about this.
7. What are your views about maternity protection benefits that should be available for women who are employed in full-term, permanent positions compared to women employed in forms of non-standard employment (part-time or contract positions, for example domestic workers)? Could you please talk about whether you think the benefits should be the same or different and your reasons for this?
8. If you think of female domestic workers specifically, what maternity protection benefits do you think they are legally entitled to, currently in South Africa? *(Prompt / probe: Do you employ a domestic worker? Do you know what benefits she would be entitled to if she did happen to fall pregnant?)*
9. Can you describe how you think the legal entitlements that female domestic workers should receive are currently being implemented? *(Probe/prompt: Do you think female domestic workers are accessing the maternity protection benefits that they should be entitled to? If yes / no, why or why not?)*
10. What type of maternity protection do you think that female domestic workers should be able to access, considering that they are a group with varying employment contracts and arrangements?
11. Based on your knowledge and experience, can you think of anything that could or should be done in terms of implementing improved access to maternity protection by female domestic workers, specifically or do you feel that this is good as it is? *(Probe/prompt: Keep same as it? Improve the implementation of the existing policy or legislation? Keep policy as is? Update existing policy / legislation to include….? Keep involvement of Department of Labour? Involve other sectors such as….?)*
